# Possible pair-graphene structures govern the thermodynamic properties of arbitrarily stacked few-layer graphene

**DOI:** 10.1038/s41598-021-02995-5

**Published:** 2021-12-03

**Authors:** Yong Sun, Kenta Kirimoto, Tsuyoshi Takase, Daichi Eto, Shohei Yoshimura, Shota Tsuru

**Affiliations:** 1 Department of Applied Science for Integrated System Engineering, Kyushu Institute of Technology, 1-1 Senshuimachi, Tobata, Kitakyushu-City, Fukuoka 804-8550 Japan; 2 Department of Electrical and Electronic Engineering, Kitakyushu National College of Technology, 5-20-1 shii, Kokuraminami, Kitakyushu-City, Fukuoka 802-0985 Japan; 3 Department of Humanities, Baiko Gakuin University, 1-1-1 Koyocho, Shimonoseki-City, Yamaguchi 750-8511 Japan

**Keywords:** Nanoscience and technology, Graphene, Thermodynamics

## Abstract

The thermodynamic properties of few-layer graphene arbitrarily stacked on LiNbO_3_ crystal were characterized by measuring the parameters of a surface acoustic wave as it passed through the graphene/LiNbO_3_ interface. The parameters considered included the propagation velocity, frequency, and attenuation. Mono-, bi-, tri-, tetra-, and penta-layer graphene samples were prepared by transferring individual graphene layers onto LiNbO_3_ crystal surfaces at room temperature. Intra-layer lattice deformation was observed in all five samples. Further inter-layer lattice deformation was confirmed in samples with odd numbers of layers. The inter-layer lattice deformation caused stick–slip friction at the graphene/LiNbO_3_ interface near the temperature at which the layers were stacked. The thermal expansion coefficient of the deformed few-layer graphene transitioned from positive to negative as the number of layers increased. To explain the experimental results, we proposed a few-layer graphene even–odd layer number stacking order effect. A stable pair-graphene structure formed preferentially in the few-layer graphene. In even-layer graphene, the pair-graphene structure formed directly on the LiNbO_3_ substrate. Contrasting phenomena were noted with odd-layer graphene. Single-layer graphene was bound to the substrate after the stable pair-graphene structure was formed. The pair-graphene structure affected the stacking order and inter-layer lattice deformation of few-layer graphene substantially.

## Introduction

Few-layer graphene, a two-dimensional (2D) carbon material with atomic thickness, has a negative in-plane thermal expansion coefficient (TEC) due to both graphene sheet rippling^1–4^ and increasing phonon out-of-plane vibrations^5–7^. This negative TEC results in many challenges in the area of electronic devices because 2D carbon materials generally require a 3D material as a substrate and most 3D materials such as Si, SiO_2_, and SiC have positive TECs near room temperature. These diametrically opposite temperature coefficients often lead to residual thermal stress at the 2D/3D material interface, which greatly affects the electrical properties of the 2D carbon material and leads to instability regarding the characteristics of the resulting electronic devices.

Graphene is a promising 2D material for electronic devices for which extensive applied research is available^8,9^. However, graphene layers must be grown on a catalyst substrate such as copper or nickel foil. It is also necessary to transfer the resulting graphene layer to a substrate in order to fabricate electronic devices. Several types of crystal defects such as point defects^10^, one-dimensional dislocations^11^ grain boundaries^12^, and wrinkles^2,13–15^ formed during growth and ripple defects from the transfer process can be introduced into the graphene layer. Defects can form during in growth due to both crystal imperfections of the catalyst substrate and large temperature differences during cooling from the growth temperature to room temperature. On the other hand, defects introduced during the transfer process, such as ripples, form at room temperature via deformation of the graphene layer^16–18^ and introduction of stress^19,20^. For above reasons, perfect or defect-free graphene layers have not yet been obtained. Therefore, it is important to elucidate the effects of defects on the TEC of a graphene layer.

It has been reported that the Young’s modulus of graphene increases as the atomic vacancy density decreases^21,22^. Other experimental results have showed that negative graphene TECs are related to in-plane contractions of ripples and wrinkles in the graphene layer^5,23,24^ Recently, molecular dynamics (MD) simulations showed that graphene origami structures obtained via pattern-based surface functionalization provide TECs that are tunable from large negative values such as − 465 × 10^−6^ to large positive values such as + 33 × 10^−6^ K^−1^ between 250 and 350 K^25^. These simulations showed that the mechanisms that give rise to this property are exclusive to graphene origami structures, as they emerge from a combination of surface functionalization, large out-of-plane thermal fluctuations, and the 3D geometries of the origami structures.

Measurement of graphene TECs is challenging because conventional experimental techniques designed for bulk materials cannot be applied to such thin samples. For this reason, few new graphene TEC measurement methods have been reported. Using atomic force microscopy (AFM), it is possible to accurately measure the indentation and stress of a suspended graphene sheet^22,26^. When a suspended sheet is pressed on using an AFM tip, its TEC can be obtained by measuring the temperature dependences of various stresses. Also, experimental estimation of the graphene TEC has been performed by analyzing the temperature-dependent shift of the Raman G band of the graphene layer on SiO_2_ while carefully excluding substrate effects^4^. However, few-layer graphene is energetically unstable and defects and ripples are introduced, especially when the layer is stacked on a 3D substrate. Many challenges remain regarding evaluation of the thermodynamic properties of such deformed few-layer graphene.

Surface acoustic wave (SAW) propagation is a powerful method of investigating the mechanical and electrical properties of low-dimensional materials such as 2D electron gases^27,28^, graphene^29,30^, 1D carbon nanotubes^31,32^, and zero D quantum dots^33,34^. In this study, we investigated the thermodynamic properties of deformed few-layer graphene by analyzing the propagation velocities, frequencies and attenuation characteristics of SAWs that passed through the graphene layers. We found that the SAWs were sensitive to changes in the number of graphene layers. Deformation of the graphene layers resulted in a large TEC change from negative to positive as the number of graphene layers increased from one to five. A stable pair-graphene structure formed preferentially in few-layer graphene. The pair-graphene structure played an important role in thermodynamic properties such as the TEC and interfacial friction of few-layer graphene.

## Results and discussion

### Few-layer graphene stacking structure

In this study, the graphene layers were transferred layer-by-layer onto the surfaces of LiNbO_3_ crystals at room temperature under atmospheric conditions. The graphene layers have an incommensurately stacked structure and there is no crystalline correlation between the layers. In order to confirm their stacking structures, the graphene/LiNbO_3_ structures were analyzed using X-ray diffraction. The X-ray measurements were performed at 45.0 kV and 200 mA, with a wavelength of 0.15406 nm and with 2θ between 5° and 85°. The wavelength of the X-ray used in this study is almost the same as the distance between adjacent carbon atoms in the graphene sheet, 0.142 nm. Thus, large X-ray reflectance occurs during the diffraction measurement.

**Figure 1:**
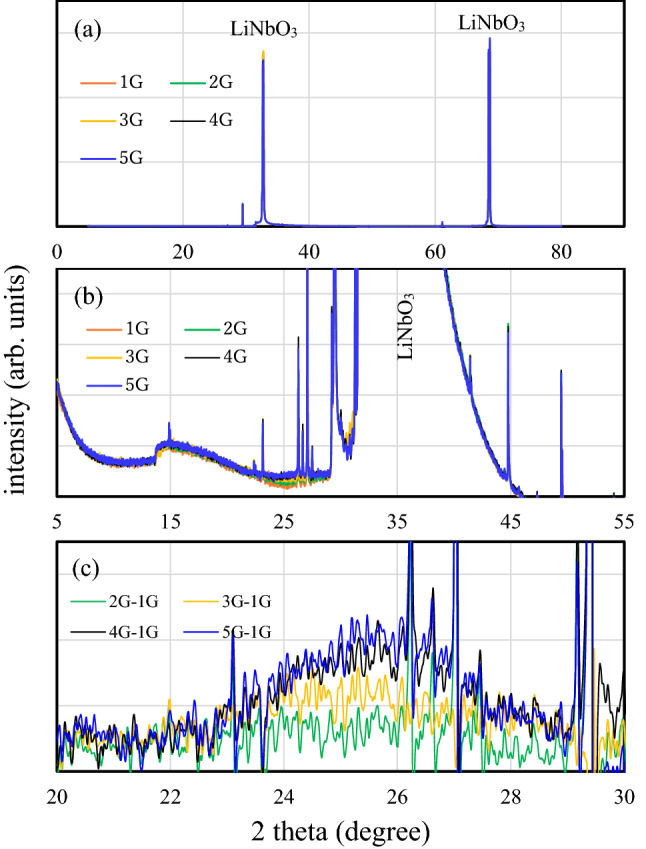
(**a**) X-ray diffraction patterns of mono-, bi-, tri-, tetra-, and penta-layer graphene/LiNbO_3_ structures with 2θ between 5° and 85°. (**b**) An enlarged view of the X-ray diffraction patterns of mono-, bi-, tri-, tetra-, and penta-layer graphene/LiNbO_3_ structures with 2θ between 5° and 55°. (**c**) Differences between the diffraction intensities of bi-, tri-, tetra-, and penta-layers and those of mono-layer sample with 2θ between 20° and 30°.

X-ray diffraction patterns of mono-, bi-, tri-, tetra-, and penta-layer graphene/LiNbO_3_ structures in the 2θ range of 5°–85° are shown in Fig. 1a. Two strong, sharp diffraction peaks are observed at 2$\uptheta =32.75^\circ$ and 2$\uptheta =68.68^\circ$, which correspond to the diffraction plane of the LiNbO_3_ crystal. An enlarged view of the X-ray diffraction patterns of mono-, bi-, tri-, tetra-, and penta-layer graphene/LiNbO_3_ structures in the 2θ range of 5°–55° are shown in Fig. 1b. In addition to the 2$\uptheta =32.75^\circ$ peak, several sharp peaks may be from the LiNbO_3_ crystal or aluminum IDT metal electrodes. It is interesting that the diffraction intensity varies with the number of layers near 2$\uptheta =25.66^\circ$. The differences between the diffraction intensities of the bi-, tri-, tetra-, and penta-layers versus the mono-layer sample are shown in Fig. 1c in the 20°–30° range. Broad diffraction peaks are observed from samples with bi-, tri-, tetra-, and penta-layers. Using results from the broad diffraction peaks, we determined the spacings of the graphene layers stacked on the LiNbO_3_ crystal. These are 0.345 nm for the tri-layer graphene and 0.335 nm for the tetra- and penta-layer samples.

Based on the above X-ray diffraction measurement results, the following facts are strongly suggested. First, the spacings of the tetra- and penta-layers are close to that of graphite, i.e., 0.335 nm. Second, the spacing of the tri-graphene layer is 0.345 nm, which is slightly larger than that of graphite. Third, the spacing of the bi-layer may be much larger than that of the tri-layer. In other words, the few-layer graphene spacing decreases as the number of layers increases. The spacing approaches that of the graphite basal plane when the number of layers is four or more. Based on the size of the graphene layer ($10 \times 10\, {\text{mm}}^{2}$) and the incommensurate transfer conditions, the few-layer graphene has an ABCDE stacking structure and no crystal relationship, where A, B, C, D and E correspond to different angles between crystal axes on each basal plane of graphene layers.

It has also been reported that the spacing of the basal plane of the nano-graphene oxide changes substantially with the crystal size and residual oxidizing agent content. The basal plane spacing of nano-graphene oxide decreases from 0.83 to 0.37 nm due to desorption of both oxygen functional groups and adsorbed gas molecules under vacuum and thermal annealing conditions^35^. A spacing decrease has also been reported upon reduction of few-layer graphene oxide. The spacing decreases from 0.9 to 0.4 nm due to desorption of functional oxygen groups and water molecules^36^.

Our X-ray diffraction measurement results indicate that the physically adsorbed gas and residual oxidizing agent molecules can be desorbed sufficiently at room temperature under vacuum conditions, and that the arbitrarily stacked graphene layers on the LiNbO_3_ crystal have spacing characteristics that are similar to that of the graphite basal plane.

### Intra-layer lattice deformation in few-layer graphene

Intra-layer lattice deformation is a strain state in which phonons do not leak to the neighboring graphene layers. The magnitude of this deformation is relatively small and the phonons remain confined in each graphene layer. Intra-layer lattice deformation can be observed by mapping Raman scattering peak shifts in the basal plane of few-layer graphene.

Graphene/LiNbO_3_ structures were characterized at room temperature in open air using a micro-Raman spectrometer with a laser wavelength of 532 nm, accuracy of 3.8 ${\text{cm}}^{-1}$, and power of 12 mW. The scanning step and 2D-mapping area were 2.0 ${\upmu m}$ and $100\times 100 {{\upmu m}}^{2}$, respectively.

Using Raman scattering measurements, many scattering peaks from the graphene layers and LiNbO_3_ crystal are confirmed in the 100–3800 cm^−1^ wavenumber range. In this study, we focus on the G band near 1581 ${\text{cm}}^{-1}$ to characterize intra-layer phonon vibration behavior in few-layer graphene because the G band is known to soften and split in graphene that is subject to uniaxial strain^37,38^. In general, the G band is sharp and appears at 1581.72 ${\text{cm}}^{-1}$ for graphite but shifts to 1584.16 ${\text{cm}}^{-1}$ for bi-layer and 1587.94 ${\text{cm}}^{-1}$ for mono-layer graphene, respectively^39^. The G band wavenumber and intensity are highly sensitive to the number of layers. The intensity can allow accurate thickness determination and follows a linear trend as one progresses from single to multilayer graphene^39^.

**Figure 2:**
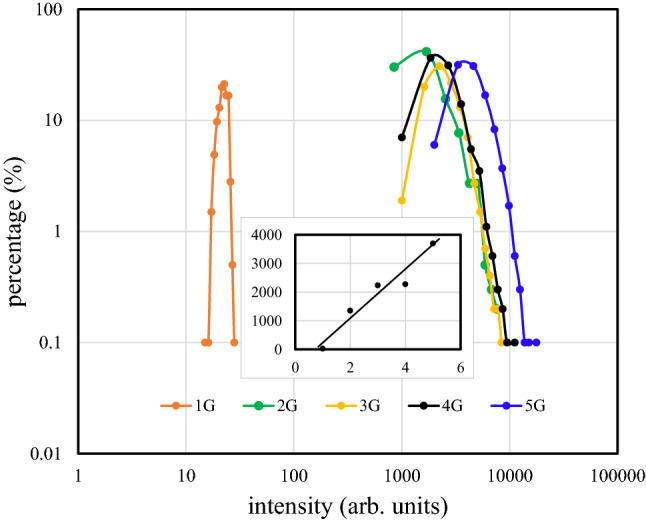
The percentages of G band intensities for mono-, bi-, tri-, tetra-, and penta-layer graphene/LiNbO_3_ structures.

Due to sources of non-uniformity within the few-layer graphene structure on the LiNbO_3_ substrate such as crystal defects, thickness distributions, residual stresses, and deformation, there is a 2D Raman scattering intensity distribution across the mapping area. The percentages of the G band intensities for graphene/LiNbO_3_ structures with mono-, bi-, tri-, tetra-, and penta-graphene layers are shown in Fig. 2. The maximum percentages for the mono-, bi-, tri-, tetra-, and penta-layer samples are centered at intensities of 23 cps, 1346 cps, 2230 cps, 2269 cps, and 3692 cps, respectively. Also, the Raman scattering intensity is shown in the inset of Fig. 2 as a function of the number of layers. This linear dependence indicates that the intra-layer phonon vibrations are mostly confined in specific graphene layers and that a weak van der Waals interlayer interaction is also present. Also, it is clear in Fig. 2 that the percentage contribution of the G band intensity is quite sharp in mono-layer graphene, but becomes broader as the number of layers increases. Specifically, the crystallinity of the mono-layer is nearly perfect but crystal defects and deformation are introduced to the stacked film as the number of layers increases.

**Figure 3:**
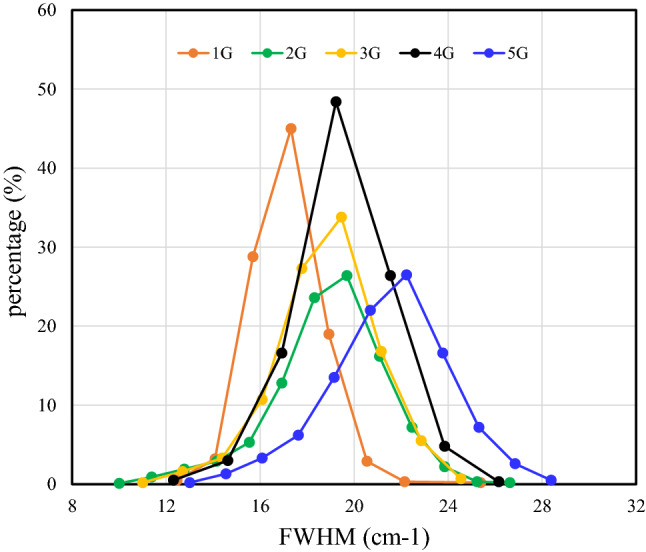
The percentages of the FWHM of the G band in the wavenumber range of 1577.00 ${\text{cm}}^{-1}$ to 1590.77 ${\text{cm}}^{-1}$.

The G band is an in-plane vibrational mode that involves the ${\text{sp}}^{2}$ hybridized carbon atoms that comprise the graphene sheet. Therefore, its full width at half maximum (FWHM) reflects the unity of the in-plane vibration frequency and perfection of the graphene lattice sheet. The percentage of the G band that falls within the 1577.00 ${\text{cm}}^{-1}$ to 1590.77 ${\text{cm}}^{-1}$ wavenumber range is shown in Fig. 3 as a function of the FWHM. It is clear that the G band widens and shifts towards higher FWHMs when the number of layers increases. The results suggest that graphene layer stacking causes dispersion in the vibration frequency of the graphene lattice and decreased crystallinity.

**Figure 4:**
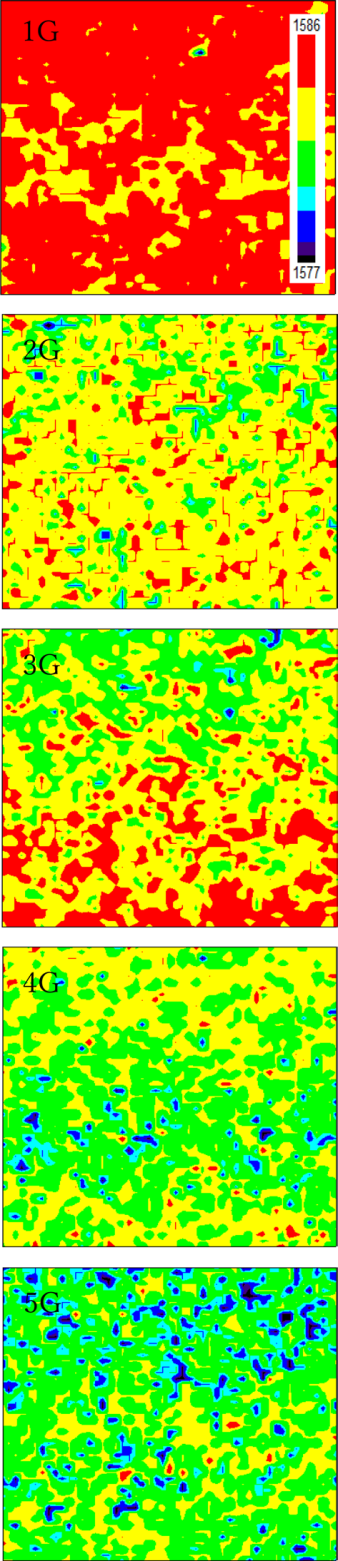
Wavenumber mapping of the G band for mono-, bi-, tri-, tetra-, and penta-layer graphene/LiNbO_3_ structures between 1577.0 ${\text{cm}}^{-1}$ and 1586.0 ${\text{cm}}^{-1}$.

In addition, the wavenumber of the G band is sensitive to graphene lattice deformation and can be detected with a high signal-to-noise ratio even when there is only a single graphene layer. The wavenumber in red-shifted when the graphene lattice is expanded, and blue-shifted when it is compressed^40,41^. G-band wavenumber mapping for mono-, bi-, tri-, tetra-, and penta-layer graphene/LiNbO_3_ structures are shown in Fig. 4 in the 1577.0 ${\text{cm}}^{-1}$ to 1586.0 ${\text{cm}}^{-1}$ range. First, the wavenumber is non-uniform when the mapping area exceeds several tens of square micrometers. Therefore, this non-uniform distribution forms due to deformation of the graphene lattice, regardless of the crystal lattice size. Second, the G-band wavenumber of the mono-layer sample is 1586.0 ${\text{cm}}^{-1}$ in almost all mapping areas, and decreases to 1577.0 ${\text{cm}}^{-1}$ as the number of layers increases among tetra-, and penta-layer samples in partial mapping areas. In fact, the G-band wavenumber is 1587.94 ${\text{cm}}^{-1}$ for a freestanding single graphene layer^39^, 1581.72 ${\text{cm}}^{-1}$ for graphite crystal^39^, and 1580.80 ${\text{cm}}^{-1}$ for the mono-graphene layer on a SiO_2_ substrate^42^. The substrate results always red-shift. This may be related to the negative charge transfer from graphene to the substrate. Negative charge transfer due to the large electron affinities of oxide substrate materials can result in changes to the electronic structure and lattice constant of few-layer graphene. Also, it is clear in the figure that the average G-band wavenumbers of tetra- and penta-layer samples are close to that of graphite, a perfect crystal. The results suggest that size of the deformed area in few-layer graphene decreases as the number of layers increases.

### Inter-layer lattice deformation in few-layer graphene

Inter-layer lattice deformation refers to a strain state in which phonons can leak into neighboring graphene layers. Information about inter-layer lattice deformation cannot be directly separated from G-band Raman shifts in the 2D mapping on the basal plane, but this information can be analyzed by measuring samples with different numbers of layers. Analysis of interactions between SAWs and graphene layers can provide important evidence regarding inter-layer lattice deformation.

**Figure 5:**
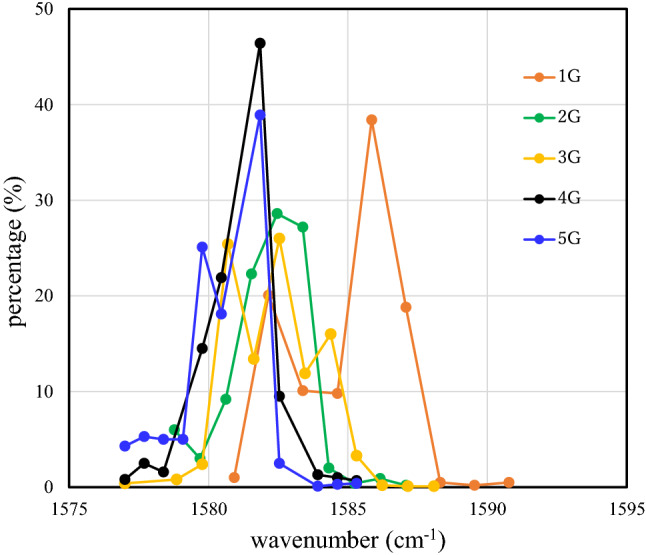
Percentage of the wavenumber of the G band for mono-, bi-, tri-, tetra-, and penta-layer graphene/LiNbO_3_ structures between 1575.0 ${\text{cm}}^{-1}$ and 1595.0 ${\text{cm}}^{-1}$.

The percentages of the G-band wavenumber in mono-, bi-, tri-, tetra-, and penta-layer graphene/LiNbO_3_ structures between 1575.0 ${\text{cm}}^{-1}$ and 1595.0 ${\text{cm}}^{-1}$ are shown in Fig. 5. In mono-, tri-, and penta-layer samples (with odd numbers of layers), the wavenumber percentage has a broad distribution and plurality of peaks can be confirmed. This result indicates strong interactions between layers and the substrate. In contrast, bi-, and tetra-layer samples with even numbers of layers exhibit wavenumber percentage distributions that are narrow and contain only a single peak. This indicates weak-interactions between the layers and the substrate. The above results show that the stacking order of few-layer graphene is controlled by the number of graphene layers. In particular, there are stronger interactions between few-layer graphene and the substrate in mono-, tri-, and penta-layer samples than in bi-, and tetra-layer samples. Stronger influence from the substrate, such as charge transfer from the layers to the substrate and the presence of defects at the substrate surface, results in inter-layer lattice deformation of few-layer graphene.

Also, inter-layer lattice deformation allows phonons to leak into neighboring graphene layers. This can be detected by measuring the parameters of SAWs that pass through the graphene/LiNbO_3_ interface. The thermal coefficient of the fundamental frequency ${f}_{0}$ (TCF) of LiNbO_3_ crystal is well known as one important parameter of the SAW device. Its value is reported to be $-\,7.3\times {10}^{-5} {\text{K}}^{-1}$ near room temperature^43^. The TCF is sensitive to presence of graphene layers because mass accumulation changes the Young’s modulus and mass density of the graphene/LiNbO_3_ interface^44^.

**Figure 6:**
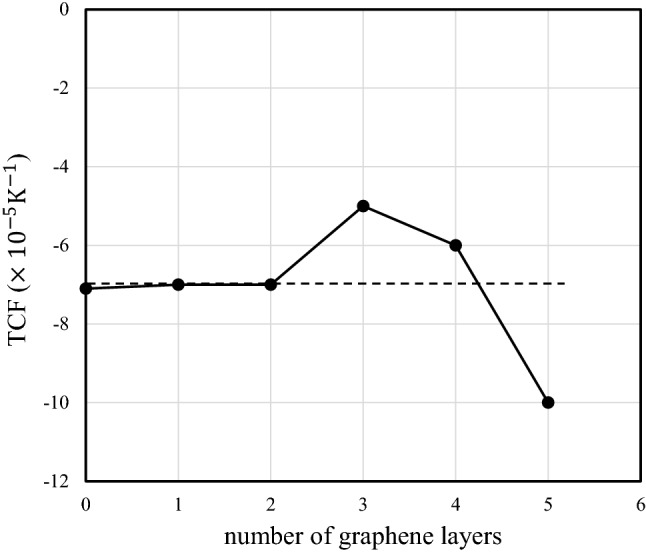
The thermal coefficients of the fundamental frequencies of SAW devices with mono-, bi-, tri-, tetra-, and penta-layers as functions of the number of layers. Here, the measurement accuracy of the TCF is $\pm \,0.1\times {10}^{-5}{\text{K}}^{-1}$.

TCFs of zero-, mono-, bi-, tri-, tetra-, and penta-layer SAW devices are shown in Fig. 6 at 280–320 K as functions of the number of layers. In the figure, the TCF of the LiNbO_3_ crystal without a graphene layer is $-\,7.1\times {10}^{-5} {\text{K}}^{-1}$, which is close to the reported value of $-\,7.3\times {10}^{-5} {\text{K}}^{-1}$^43^. It is clear that the TCFs of zero-, mono-, bi-, and tetra-graphene layer samples are almost the same, but the TCFs of the tri- and penta-layer samples are higher and lower, respectively. The different TCFs of the tri- and penta-layer samples indicate strong interactions between graphene layers and phonons leakage into neighboring graphene layers. The above results indicate that the Young’s modulus of few-layer graphene varies when the number of layers is odd.

**Figure 7:**
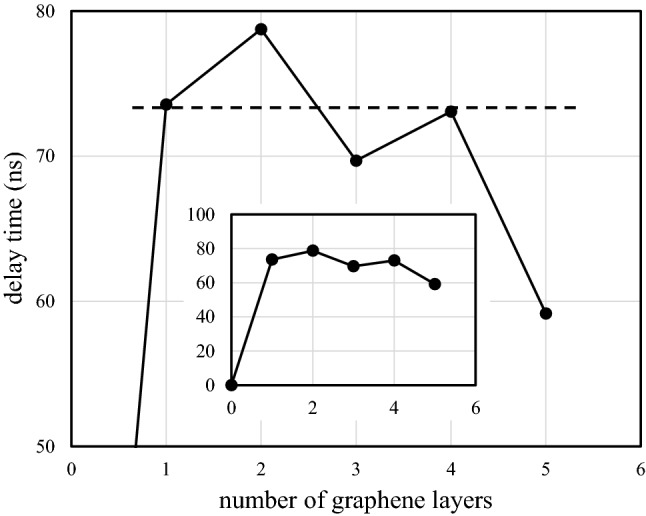
The relative delay times for SAWs that pass through the delay lines on LiNbO_3_ crystals with mono-, bi-, tri-, tetra-, and penta-graphene layers as functions of the number of graphene layers. Here, the measurement accuracy of the delay time is $\pm$ 1 ns.

Moreover, inter-layer lattice deformation can be confirmed by measuring the propagation velocity of a SAWs that passes through the graphene/LiNbO_3_ interface. The relative delay times of SAWs that pass through zero-, mono-, bi-, tri-, tetra-, and penta-layer graphene/LiNbO_3_ interfaces are shown in Fig. 7 as functions of the number of layers. These delay times are relative to the delay time of the SAW device without the graphene layer, which is shown in the inset of the figure. The delay times of the tri- and penta-layer samples are smaller than those of the mono-, bi-, and tetra-layer samples. This indicates that a large inter-layer interaction, namely lattice deformation, occurs in the tri- and penta-layer samples. In other words, the smaller delay time corresponds to a larger propagation velocity and indicates that the phonons flow out from each layer of the deformed few-layer graphene. This corresponds to an even–odd layer number effect.

Two inter-layer phonon interaction models or inter-layer vibrational models have been reported. Low-energy inter-layer vibrations comprise layer breathing modes^45–47^ and shearing modes^48^. These modes involve the relative displacement of individual graphene layers in the in-plane and out-of-plane directions, respectively. Based on nearest-neighbor coupling between the graphene layers, these vibrational modes can be observed in few-layer graphene with two or more layers. In N-layer graphene, the layer breathing vibrations create a set of N-1 out-of-plane modes. However, there is no even–odd layer number effect on the above layer breathing and shearing modes. Therefore, these vibration modes are not the dominant mechanism of inter-layer deformation in few-layer graphene.

### Few-layer graphene stacking order

The above results confirm that, in addition to van der Waals interactions, phonon interactions occur between graphene layers in few-layer graphene with odd layer counts. In fact, a pair-graphene structure with a more stable energy state has been identified in few-layer graphene^49–53^. In few-layer graphene, there is an even–odd layer number effect: the band structures of 2 N (N = 1, 2, 3,…) layer graphene exhibits N bilayer-like bands, and 2 N + 1 layer graphene additionally exhibits a monolayer-like band. Such structural effects result in a unique stacking order in the few-layer graphene. Therefore, we can propose the following stacking order model: I/for mono-, II/for bi-, II/I/for tri-, II/II/ or tetra-, and II/II/I/for penta-layer graphene/LiNbO_3_, where I/ represents a single layer and II/ represents paired layers. In samples with odd numbers of layers, the single layer binds to the substrate and paired-layers form on the single layer because such a stacking order is energetically stable. Since the single layer has a linear dispersion relation and a bandgap of zero, charge transfer to the substrate is easier. In contrast, charge transfer is hindered by a non-zero bandgap, resulting in weaker interactions between graphene layers with even numbers of layers and the substrate. It has been reported that the bandgap of two-layer graphene varies with charge transfer and that the range of variation is on the order of several hundred meV^51,52^.

Also, because of its high carrier mobility, the screening length of graphene is reported to be comparable to the thickness of a single graphene layer^50,54^. Therefore, once a single graphene layer is transferred to the LiNbO_3_ substrate, the electronic and electrical influences of the substrate can be nearly blocked. After the first layer, the even–odd layer number effect dominates stacking order formation in few-layer graphene.

In addition to the effects from charge transfer and substrate defects, external electric fields also affect the energy bandgap of few-layer graphene^55–57^. However, energy bandgap variation can be ignored when the electric field strength is less than ${2\times 10}^{-2 }\text{V} \text{nm}^{-1}$^56,57^. In this study, the maximum value of the piezoelectric potential electric field components in the parallel and vertical directions on the LiNbO_3_ surface is $7\times {10}^{-4 }\text{V} \text{nm}^{-1}$, so the effect on the energy band structure is sufficiently small.

### Stick–slip friction at graphene/LiNbO_3_ interface

Because the graphene/LiNbO_3_ structures are formed by transferring graphene layers to the LiNbO_3_ substrate, there is no thermal stress at the interface at the transfer temperature. Static friction occurs at the interface when the temperature subsequently changes. If the temperature change is sufficiently large, sliding friction appears and stick–slip behavior occurs^58^. In fact, we reported abnormal behavior regarding parameters of SAWs that pass through the penta-layer graphene/LiNbO_3_ interface in our previous research^30^. Discontinuous changes occur in both the SAW intensity and frequency near the transfer temperature. In this study, these discontinuous changes are also observed from the tri-layer graphene/LiNbO_3_ structure.

**Figure 8:**
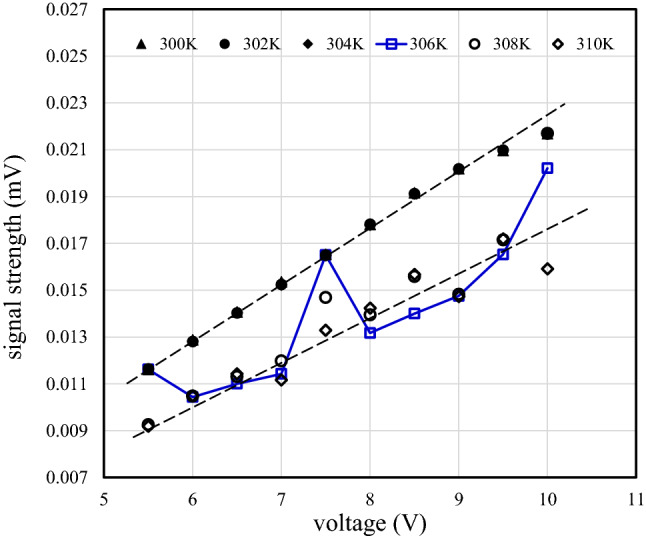
The signal strength of the output IDT electrode of a SAW device with a tri-graphene/LiNbO_3_ structure at temperatures of 300 K, 302 K, 304 K, 306 K, 308 K, and 310 K as a function of the DC voltage of the input IDT electrode.

The strengths of SAW device output signals with the tri-layer graphene/LiNbO_3_ structure at temperatures of 300 K, 302 K, 304 K, 306 K, 308 K, and 310 K are shown in Fig. 8 as function of the input DC voltage. The output signal strength is calculated from the integral of the Fourier-transformed output signal over frequency and the DC voltage is the amplitude of the input DC pulse signal. At temperatures below 306 K, such as 300 K, 302 K, and 304 K, the output signal strength increases in proportion to the input signal strength with a gradient of $2.89\times {10}^{-3}{\text{ mV V}}^{-1}$. In contrast, at temperatures above 306 K, such as 308 K and 310 K, the output signal strength increases in proportion to the input signal strength with a gradient of $2.00\times {10}^{-3} {\text{mV V}}^{-1}$. At 306 K, the output signal strength is unstable and varies between the two aforementioned gradients. This instability is observed between 305 and 307 K. The output signal strength varies between measurements within this temperature range.

**Figure 9:**
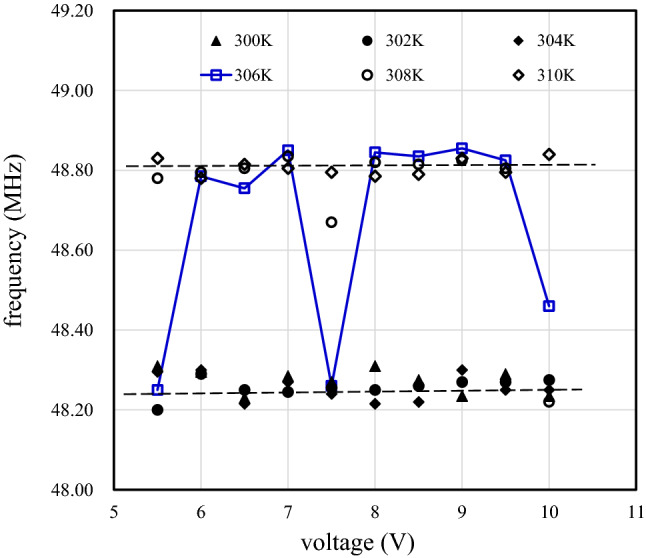
The fundamental frequencies of a SAW device with a tri-graphene/LiNbO_3_ structure at temperatures of 300 K, 302 K, 304 K, 306 K, 308 K, and 310 K as functions of the DC voltage of the input IDT electrode.

Moreover, the fundamental frequencies $f$ of the SAW device with tri-layer graphene at temperatures of 300 K, 302 K, 304 K, 306 K, 308 K, and 310 K are shown in Fig. 9 as functions of the input DC voltage. At temperatures below 306 K, such as 300 K, 302 K and 304 K, $f$ has an average value of 48.245 MHz. This value varies little and does not depend on the input DC voltage. On the other hand, at temperatures above 306 K, such as 308 K and 310 K, $f$ has an average value of 48.815 MHz. This value also varies little and does not depend on the input DC voltage. At 306 K, $f$ is unstable and varies between 48.245 and 48.815 MHz. As with the output signal strength, $f$ varies between measurements when the temperature is between 305 and 307 K.

The above results indicate that the output signal strength is proportional to the input signal strength, and that $f$ does not depend on the input signal strength. Thus, the SAW device is working normally. Second, the instabilities of the signal strength and $f$ show that temperature-dependent stress is formed at the graphene/LiNbO_3_ interface. It acts as a compressive stress as the temperature increases, resulting in an increase in $f$. Third, the temperature at which the instability occurs corresponds to the graphene layer transfer temperature^30^. Fourth, temperature-dependent stress occurs because of interfacial stick–slip friction that is dependent on the graphene and LiNbO_3_ crystal lattice structures. The difference between the graphene and LiNbO_3_ lattice constants can lead to the appearance of moiré patterns with larger periodic structures than those of graphene and LiNbO_3_^59,60^. The stick–slip friction observed in this study may be related to the moiré pattern period and occurs within $\pm 1$ K of the transfer temperature (306 K). Fifth, the stick–slip friction observed with tri- and penta-layer graphene samples indicates that the presence of relatively large static friction although the associated sliding friction is nearly zero^61^. We also must point out that the single graphene layer bonded to the LiNbO_3_ surface plays an important role in the stick–slip friction process.

### Thermal expansion coefficients of deformed few-layer graphene

**Figure 10:**
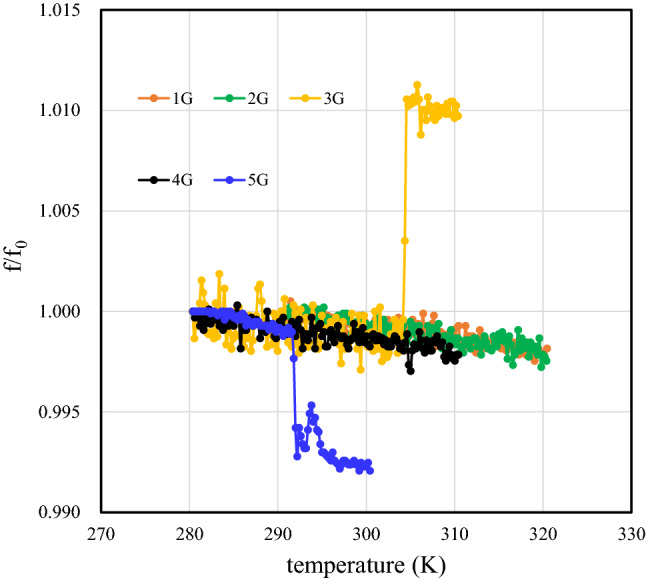
The fundamental frequencies of a SAW device with mono-, bi-, tri-, tetra-, and penta-layers as functions of the temperature. Here, the frequencies are divided by the ${f}_{0}$ of the penta-layer sample at 280 K.

The fundamental frequency ratios $f/{f}_{0}$ of SAW devices with mono-, bi-, tri-, tetra-, and penta-layer graphene are shown in Fig. 10 as functions of the temperature. Here, ${f}_{0}$ is the fundamental frequency of the penta-layer sample at 280 K. The $f/{f}_{0}$ ratio decreases as the temperature increases. The TCF is negative between 280 and 320 K, it is in agreement with the results in Fig. 6. Also, discontinuous changes in $f$ are observed at 306 K for the tri-layer sample and at 292 K for the penta-layer sample. The temperature range of the discontinuous change is approximately 2 K^30^. The discontinuous changes correspond to thermal contraction and expansion stresses at the LiNbO_3_ surface for the tri- and penta-layer samples, respectively. The results indicate that changing the number of layers from three to five changes the TEC from positive to negative. The results also suggest that the penta-layer sample II/II/I/ stacking order causes different inter-layer lattice deformation than the tri-layer sample II/I/ stacking order.

Also, the II/II/ tetra-layer and II/ bi-layer stacking orders (even numbers of layers) do not cause inter-layer lattice deformation and no discontinuous TEC change is detected. Moreover, the aforementioned discontinuous change is not detected in the mono-layer sample. This may be related to superlubricity^61–63^ at the graphene/LiNbO_3_ interface. A vanishing stick–slip friction phenomenon has been observed in few-layer graphenes^61–65^. Friction decreases with decreasing the number of layers and finally approaches zero for one and two graphene layers. This phenomenon has been explained quantitatively using a classic theoretical model that considers the lateral stiffness, slip length, and maximum lateral force^61^.

From the above results, it is clear that both the number of pair-graphene structures and the presence of a single layer bonded to the LiNbO_3_ substrate play important roles in both deformation of few-layer graphene and changes in its TEC.

## Conclusions

Few-layer graphene was transferred onto the surface of LiNbO_3_ crystal and stacking order of the layers was analyzed via X-ray diffraction and Raman scattering. Moreover, surface acoustic waves were propagated along the graphene/LiNbO_3_ interface to characterize its thermodynamic properties.

First, few-layer graphene was arbitrarily stacked on the LiNbO_3_ crystal surface in parallel. The tetra- and penta-layer graphene spacings were 0.335 nm, which were similar to that of graphite. The spacing of the tri-layer graphene was 0.345 nm.

Second, mono-, bi-, tri-, tetra-, and penta-layer graphene/LiNbO_3_ structures exhibited negative charge transfer from the graphene layers to LiNbO_3_ crystal because a red-shift of the Raman scattering G band was observed for all samples.

Third, the wavenumber mapping measurements of the Raman scattering G band at the basal plane indicate the presence of intra-layer lattice deformation with an average size of hundreds of square micrometers.

Fourth, measurements of both the frequency thermal coefficient of the LiNbO_3_ crystal with graphene layers and the delay times of SAWs that passed through the graphene/LiNbO_3_ interface showed that inter-layer lattice deformation occurs in few-layer graphene with odd numbers of layers.

Fifth, inter-layer lattice deformation in few-layer graphene with odd numbers of layers results in both stick–slip friction at the graphene/LiNbO_3_ interface and a TEC that transitions from negative to positive as the number of layers increases.

Finally, inter-layer lattice deformation may be related to the presence of both a stable pair-graphene structure and a single graphene layer bonded to the LiNbO_3_ surface.

## Methods

The SAWs were generated and received using interdigital transducer (IDT) electrodes on the surface of 128 degree Y-cut LiN_b_O_3_ crystal with dimensions of $10 \times 30\times 0.5 \,{\text{mm}}^{3}$. Few-layer graphene film with dimensions of $10 \times 10\, {\text{mm}}^{2}$ was transferred to the crystal surface between the IDT electrodes at room temperature. When a DC pulse voltage signal with rise time of 10 ns was introduced to the input IDT electrode, pulsed SAWs propagated along the graphene/LiN_b_O_3_ interface and finally were received as alternating pulse signals through the output IDT electrode.

The fundamental frequency of the SAW device was 50 MHz and the SAW propagation time from the input electrode to the output electrode, i.e., the delay time associated with passing through the delay line, was $4.5 {\upmu}\text{s}$. This corresponded to the IDT distance of 16 mm. Detailed information on SAW devices and signal processing techniques was reported in our previous paper^30^. The SAW fundamental frequency, attenuation, and delay time were measured to characterize interactions of the SAWs with the few-layer graphene films.

Graphene films with mono-, bi-, tri-, tetra-, and penta-layers were transferred onto the surfaces of the SAW devices at room temperature. Before the transfer process, the graphene layer was grown via CVD on a copper foil substrate with a thickness of 35 ${\upmu m}$ and purity above 99.9%. The graphene layers had sheet resistances of 1500 $\Omega /\square$ (mono-layer graphene) and 500 $\Omega /\square$ (penta-layer graphene), which corresponded to a carrier concentration of ~ 10^11^ cm^−2^.

The graphene/LiN_b_O_3_ sample was placed in a vacuum chamber with a residual gas pressure of less than 1.1 × 10^−7^ Pa. The sample temperature was controlled using a controller (LAKE SHORE, 331) and a cryostat (PASCAL CO. LTD., PASCAL-101E-N) in the range of 280–320 K in 0.1 K steps at a rate of 0.14 $\text{K }{\text{min}}^{-1}$ so that measurements could be performed during heating and cooling. A signal generator (AGILENT, 33220A) was used to generate DC pulse signal with an amplitude of 20 V. The input and output signal patterns were recorded using a high-definition oscilloscope (TELEDYNE LECROY, HDO 4104). The signal patterns recorded were analyzed using SciLab computer software in order to determine the SAW frequency, attenuation, and delay time before and after the SAWs passed through the graphene/LiNbO_3_ interface under various experimental conditions.

Graphene films on LiNbO_3_ crystals were characterized via X-ray diffraction (RIGAKU SmartLab R&D 100) and Raman scattering (JASCO NRS-5500) after the SAW measurements were performed.

## Data Availability

All data generated or analysed during this study are included in this published article.
